# Prognostic factors and scoring systems associated with outcome in pediatric acute liver failure

**DOI:** 10.1186/s12887-022-03574-x

**Published:** 2022-08-31

**Authors:** Priya Walabh, Anja Meyer, Tim de Maayer, Porai N. Moshesh, Ibrahim E. Hassan, Pravina Walabh, Christina Hajinicolaou

**Affiliations:** 1 Department of Paediatrics and Child Health, School of Clinical Medicine, Faculty of Health Sciences, University of the Witwatersrand, Princess of Wales Terrace, Parktown, Johannesburg, 2193 South Africa; 2 Paediatric Gastroenterology, Hepatology and Nutrition Unit, Charlotte Maxeke Johannesburg Academic Hospital, University of Witwatersrand, Johannesburg, South Africa; 3 Gauteng Provincial Solid Organ Transplant Division, Johannesburg, South Africa; 4 Department of Surgery, Charlotte Maxeke Johannesburg Academic Hospital, University of Witwatersrand, Johannesburg, South Africa; 5 Paediatric Gastroenterology, Hepatology and Nutrition Unit, Rahima Moosa Mother and Child Hospital, University of Witwatersrand, Johannesburg, South Africa; 6 Paediatric Intensive Care Unit, Nelson Mandela Children’s Hospital, Johannesburg, South Africa; 7 Bachelor of Science, University of Cape Town, Cape Town, South Africa; 8 Paediatric Gastroenterology, Hepatology and Nutrition Unit Head, Department of Paediatrics and Child health, Chris Hani Baragwanath Academic Hospital, University of Witwatersrand, Johannesburg, South Africa; 9 Head of Division of Paediatric Gastroenterology, Faculty of Health Sciences, University of the Witwatersrand, Johannesburg, South Africa

**Keywords:** Acute liver failure, Complications of liver transplantation, Pediatric end-stage liver disease score, Pediatric liver transplantation, Hepatitis A virus, Liver injury unit score

## Abstract

**Background:**

Pediatric acute liver failure (PALF) is an uncommon, devastating illness with significant mortality. Liver transplantation remains the mainstay of treatment for irreversible PALF. The purpose of this study was to determine the etiology and prognostic factors associated with outcome of PALF in South Africa and to evaluate prognostic scoring systems used.

**Methods:**

Records of 45 pediatric patients younger than 16 years of age who presented with PALF from 1 January 2015 till 31 October 2020 were analysed. Patients were divided into two groups with one group consisting of patients with spontaneous recovery of the liver with supportive treatment (6/45:13.3%) and the second group consisting of patients with poor outcomes who demised (19/45: 42%) or underwent liver transplantation (20/45: 44%).

**Results:**

The median age of presentation was 3.3 years (IQR 1.8–6.9) with the 1–5 years age group constituting majority of patients (55.6%). Median time to follow up was 6.1 months (IQR 0.2–28.8). Higher liver injury unit scores were observed in patients who had poorer outcomes (*P* = 0.008) with a threshold of greater than 246 having a sensitivity of 84% and specificity of 83% (*P* < 0.001). Higher peak PELD/MELD (*P* = 0.006) and admission UKELD (*P* = 0.002) scores, were found in patients with poorer outcomes. Kings College Hospital criteria (KCHC) was useful in predicting which patients would die without liver transplantation (*P* = 0.002). Liver transplantation was performed in 20/45 (44%) patients with a post transplantation 1 year patient and graft survival of 80%.

**Conclusion:**

Although, survival of PALF patients was lower than high and other low-middle income countries, outcomes post transplantation were good. Our study demonstrates the utility of dynamic scoring systems in PALF patients, it underscores the need for early referral and clinical monitoring in a tertiary center once the criteria for PALF have been met.

**Supplementary Information:**

The online version contains supplementary material available at 10.1186/s12887-022-03574-x.

## Introduction

Pediatric acute liver failure (PALF) is a well- defined yet uncommon clinical syndrome of hepatic injury with significant mortality [1–5]. There are currently no database registries in South Africa (SA) for PALF patients and therefore there is a paucity of information available on the subject [6, 7].

The etiology of PALF depends on the age and geographical location of children [8, 9]. Metabolic conditions and indeterminate hepatitis are more common in high-income countries (HIC) whereas viral causes like Hepatitis A virus (HAV) are the predominant cause for PALF in low-middle income countries (LMIC) [8, 10]. Hepatitis A induced acute liver failure (ALF) is markedly lower in countries with routine HAV immunization [10, 11].

Currently liver transplantation is the treatment of choice for irreversible PALF [12, 13]. Adequate assessment and early referral to a transplant center is vital for patients with PALF where they can be optimally managed, urgently listed and transplanted. It is extremely difficult to predict which patients would spontaneously recover with their native liver with supportive care and which would go onto have fatal consequences if not transplanted.

Existing scoring systems, such as the Kings College Hospital Criteria (KCHC), pediatric- end stage liver disease (PELD), model for end stage liver disease (MELD) and the Clichy scoring system are currently used in pediatric hepatology and transplant units [14]. None of these scoring systems have proved to be reliable predictors of survival with their native liver in PALF patients. No consensus regarding any of the scoring systems, predicting outcome in PALF have been reached [15, 16]. The pediatric liver injury units (LIU) score may also be a useful dynamic scoring system in PALF to predict outcomes but is currently not being utilized clinically [5, 16]. The aim of our study was to determine the etiology and prognostic factors associated with outcome of PALF in South Africa and evaluate the different scoring systems in our patient population in predicting outcomes.

## Patient and methods

### Study population

All pediatric patients from birth to 16 years of age with ALF as defined by the Pediatric acute liver failure study group (PALFSG) definition, referred to Charlotte Maxeke Johannesburg Academic Hospital (CMJAH), a tertiary academic hospital situated in Johannesburg in Gauteng Province in S.A, were included in the study. All patients who presented to and were referred to CMJAH as possible transplant candidates with PALF between 1 January 2015 and 31 October 2020 were included in the study. This included 45 pediatric patients. Patients with acute on chronic liver failure were excluded from the study. Patients underwent liver transplantation at the transplant centre, Wits Donald Gordon Academic Hospital (WDGMC), a private academic hospital also situated in Johannesburg. WDGMC have transplant surgeons who perform liver transplantation on pediatric public sector patients with government funding as a result of a public-private partnership. Approval for the study was obtained from Human Research Ethics Committee at University of Witwatersrand (Medical) M201176.

### Study procedures

The definition of acute liver failure according to the PALFSG includes biochemical evidence of liver injury with no evidence of chronic liver disease, hepatic-based coagulopathy not corrected by parenteral administration of vitamin K and hepatic encephalopathy present if the uncorrected international normalised ratio (INR) was > 1.5 to 1.9, but not required if INR was greater than or equal to 2 [1, 5, 17].

Investigations for pediatric patients with acute liver failure included relevant history, examination, viral studies, metabolic, autoimmune screens as well as routine biochemical tests. Basic liver failure treatment was instituted by the referring hospital in consultation with the gastroenterology team at CMJAH which included oral lactulose, empiric third generation cephalosporins, antifungals, acyclovir (herpes simplex treatment), proton pump inhibitor, intravenous vitamin K, fresh frozen plasma (FFP) if bleeding and mannitol if signs or suspicions of raised intracranial pressure. N-acetylcysteine infusion was commenced in patients with paracetamol ingestion, toxin ingestion or if the etiology was unknown.

Patients were directly referred to the transplant centre from February 2018 if Kings College Hospital Criteria (KCHC) were fulfilled. Prior to this, patients were first assessed and worked up at CMJAH before being placed on the transplant wait-list and listed as status 1A for an urgent deceased donor liver transplant. In conjunction with this, suitable living related donors were actively worked up as potential donors for the patient once they were placed on the transplant wait-list. KCHC for paracetamol-associated ALF was defined as a pH less than 7.3 or arterial lactate greater than 3.0 mmol/L(after adequate fluid resuscitation), serum creatinine greater than 300 *μmo* l/L, grade 3 or 4 hepatic encephalopathy and INR greater than 6.5; and for non-paracetamol-associated ALF it was defined as INR greater than 6.5, any grade of hepatic encephalopathy or any three of the following: age less than 10 years, unfavourable causes (Non-A, Non-B hepatitis, drug induced or indeterminate etiology), time from jaundice to encephalopathy > 7 days), INR greater than 3.5 or serum bilirubin greater than 300 *μmol* /L. [16] Prior to March 2018, no ABO incompatible liver transplants were performed at the transplant centre on pediatric patients younger than 16 years of age.

### Data collection

Data collected included demographic characteristics including age, gender, time to presentation, etiology and biochemical parameters like INR, bilirubin, lactate, ammonia levels, time to transplantation, type of donor used, outcome of transplantation and medical and surgical complications of transplantation. The PELD score was used for all patients less than 12 years of age and MELD score for patients older than 12 years of age. Using data available, PELD/MELD scores which included albumin, bilirubin, INR, growth parameters and creatinine were calculated both at admission and at the peak of the condition (reflected by the highest bilirubin and INR) using the online calculator available at https://www.mdcalc.com. United Kingdom end stage liver disease (UKELD) scores were calculated at admission and at the peak of the condition (reflected by the highest INR and bilirubin**)** using INR, Creatinine, Bilirubin and sodium also using the online calculator at https://www.mdcalc.com. The Liver injury units (LIUs) score was calculated using the following formula: (3.507 x peak total bilirubin + 45.51 x peak INR + 0.254 x peak ammonia) [18].

The National Health laboratory service (NHLS) and Lancet laboratory service were utilized by patients in the cohort.

All 45 patients were divided into two groups according to outcome with one group constituting those patients who recovered with spontaneous recovery of their native liver with supportive care only and another group of patients who demised or were transplanted and were considered as having a poor outcome.

### Data analysis

Categorical variables were described using frequencies and proportions. Pearson’s Chi squared test was used to compare proportions, otherwise Fisher’s exact test where data was sparse. Continuous variables were described using the mean and standard deviation or medians and interquartile range for non-normally distributed variables. Means and medians were compared between outcome groups using the t-test or the Wilcoxon rank sum test respectively. A receiver operating characteristic (ROC) curve analysis was used to predict thresholds for specific laboratory parameters that yielded the most accurate results for predicting worse outcomes (death or receiving a liver transplant) among study participants. Analyses were done in Stata 14, and statistical significance was set at 5%.

## Results

### Demographic characteristics

Records of 45 patients were analysed. The median age of patients with ALF was 3.3 years (IQR: 1.8–6.9) with the 1-5 yr age group constituting the majority of patients: 25/45 (55.6%), infants, 5/45 (11.1%) and children older than 5 years of age making up 15/45 (33.3%) of patients. Age was not associated with any of the outcomes. The median weight at presentation was 16 kg (IQR: 10–20) and median time of follow up was 6.1 months (IQR 0.2–28.2). There were more male than female patients at 53.3 and 46.7% respectively and black patients made up the majority at 39 (86.7%). Four (8.9%) mixed race patients, and one Indian and one white patient made up the remainder of patients at 2.2% each.

### Etiology

**Fig. 1:**
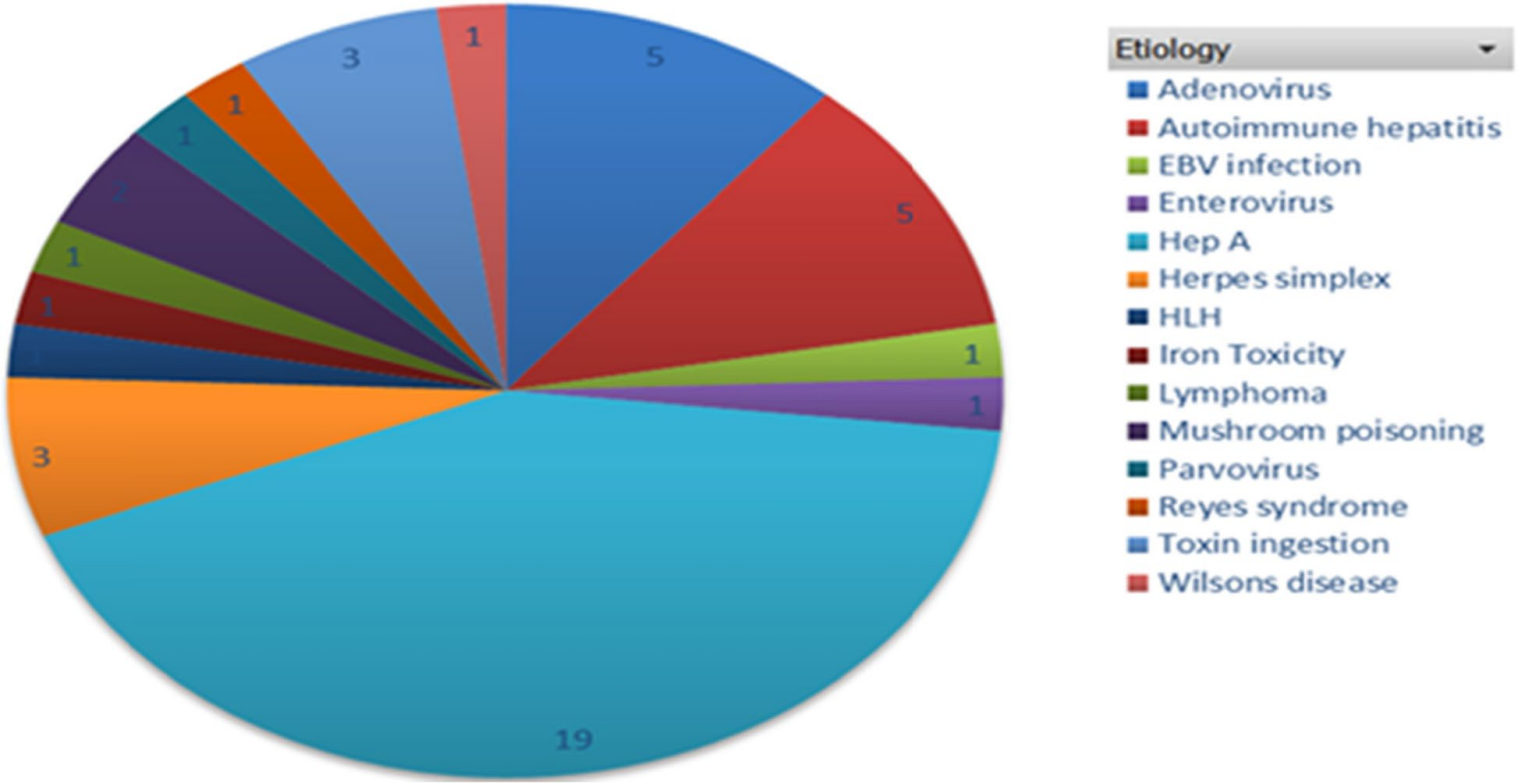
Etiology of pediatric acute liver failure patients referred to Charlotte Maxeke Johannesburg Academic Hospital (Tertiary Academic Hospital)

Thirteen percent (6/45) of patients had spontaneous recovery of the liver while 44% (20/45) were transplanted and the rest (19/45: 42%) died before transplantation. Most patients were found to have a viral etiology; 66.7% (30/45) with HAV accounting for 63.3% (19/30) of the viral cases and 19/45 (42%) of all cases of PALF in our cohort. Drug/toxins made up 13.3% of cases with metabolic and other causes making up the rest (Fig. 1). Hepatitis A was not associated with outcome in our cohort. {Unadjusted OR = 1.55, 95% CI [0.25–9.46], *P* = 0.638}.

### Prognostic indicators

Higher peak INR (*P* = 0.03), peak bilirubin levels (*P* = 0.04) and peak ammonia levels (*P* = 0.005) were found in PALF patients with poorer outcomes. Lower glucose (*P* = 0.04) and fibrinogen (*P* = 0.008) levels at referral were associated with poorer outcomes as were higher lactate levels (*P* = 0.002) (Table 1). Peak INR was found not to be significantly raised in patients who died prior to transplantation compared to those that were transplanted or recovered (*P* = 0.078) (Table 1). Patients who died prior to transplantation were found to have raised alpha-fetoprotein levels (*P* = 0.027) and lower phosphate levels (0.033) compared with patients who were transplanted or survived without transplantation (Table 1).

**Table 1:** Biochemical Parameters of patients referred with pediatric acute liver failure

Variable	Medians and IQR						
	Total ***N*** = 45	Recovered (***n*** = 6)	Died or transplanted (***n*** = 39)	***P*** value^**a**^	Transplanted patients (***n*** = 20)	Died before transplant (***n*** = 19)	***P*** value^**b**^
**Peak INR**	7.2 (4.5–10.0)	3.7 (2.2–4.7)	7.6 (4.7–10.0)	**0.030**	7 (3.9–10.0)	7.8 (5.3–10.0)	0.078
**Fibrinogen (g/L)**	1.1 (1.0–1.4)	1.6 (1.2–2.0)	1.0 (1.0–1.3)	**0.008**	1.1 (1.0–1.4)	1.0 (0.9–1.2)	**0.020**
**Lactate (mmol/L)**	4 (3–5)	2.6 (2–3)	4 (3–5)	**0.002**	4.5 (4–5.8)	4 (3–4.5)	**0.002**
**Glucose at presentation (mmol/L)**	3.5 (2.5–5.5)	5.9 (5–7)	3.1 (2.5–5)	**0.044**	2.5 (2.0–4.2)	3.6 (3.0–5.5)	**0.016**
**AFP (μg/L)**	72.6 (9.7–707)	56.4 (6.9–7186)	72.6 (9.7–707)	0.894	10.0 (7.7–704)	111.5 (46–823)	**0.027**
**Peak ammonia (μmol/L)**	154 (108–189)	82 (60–108)	162 (120–208)	**0.005**	159 (120–290)	168.5 (116–184.5)	**0.017**
**Factor 5 (%)**	23 (12–34)	125 (8–127)	22.5 (16–32)	0.435	25 (20–31)	21 (12–34)	0.734
**Albumin (g/L)**	26 (22–31)	29 (28–35)	25 (22–31)	0.136	24 (22–31)	26 (23.5–29.5)	0.286
**Bilirubin Peak (IU/ml)**	307 (82–398)	68.5 (30–322)	320 (211–425)	**0.037**	218 (76–365)	353.5 (313.5–522)	**0.002**
**Phosphate (mmol/L)**	1.1 (0.9–1.4)	0.9 (0.8–1.3)	1.2 (0.9–1.6)	0.236	1.4 (1.1–2.3)	0.9 (0.9–1.2)	**0.033**

*Abbreviations: INR* International normalized ratio, *AFP* Alpha-fetoprotein, *Factor V* Factor five

*p* value^a^ compare recovered versus demised or transplanted patients, *p* values^b^ compare laboratory parameters between three groups i.e., recovered, transplanted and died before transplant

**Fig. 2:**
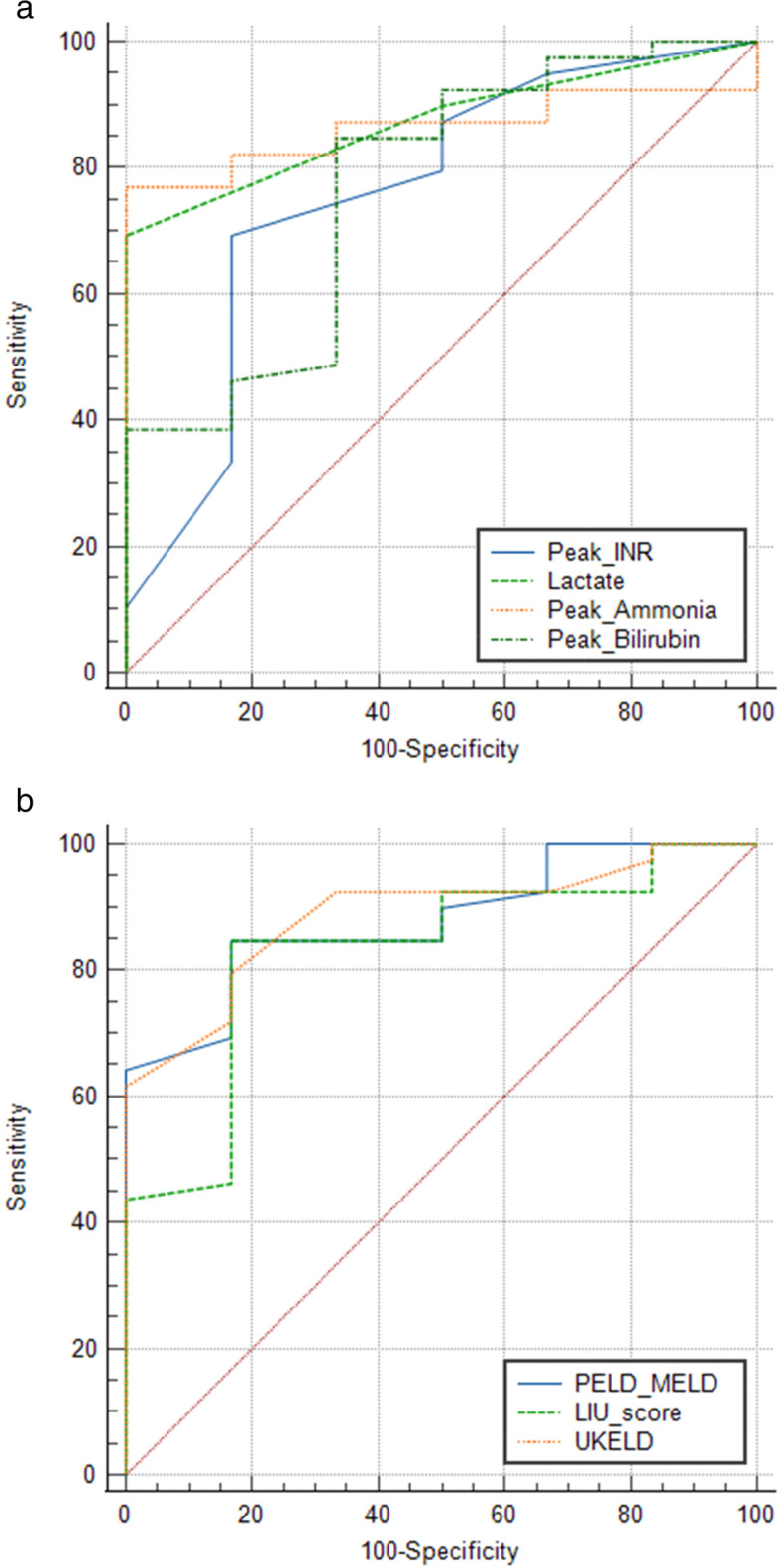
**a** Receiver operating characteristics (ROC) curve comparing biochemical parameters in pediatric acute liver failure patients with poor outcomes. Peak INR > 5; sensitivity 69% and specificity 83%; {AUC 0.76, *P* < 0.03}. Lactate > 3.0 mmol/l; sensitivity 69% and specificity 100%; {AUC 0.87, *P* < 0.0001}. Peak ammonia > 115 μmol/l; sensitivity 76.9% and specificity 100%; {AUC 0.86, *P* < 0.001}. Peak Bilirubin > 77 mmol/l; sensitivity 84.6% and specificity 66.7%; {AUC 0.77, *P* < 0.02}. **b** Receiver operating characteristics (ROC) curve comparing scoring systems in pediatric acute liver failure patients with poor outcomes. Peak Peld-Meld > 29; sensitivity of 85% and specificity of 83%; {AUC 0.88, *P* < 0.001}. LIU score > 246; sensitivity of 84% and specificity of 83%; {AUC 0.83, *P* < 0.001}. UKELD score > 63; sensitivity 80% and specificity 83%; {AUC 0.89, *P* < 0.0001}

A cut off ammonia level of 115 *μmol*/*l* using the ROC curve showed a specificity of 100% and a sensitivity of 77% for poorer outcome [PPV 100% and NPV 65%, AUC 0.86 95% CI (0.73–0.95) *P* < 0.001]. A cut off lactate level of 3.0 mmol/l using the ROC curve showed a specificity of 100% and a sensitivity of 69% for poorer outcomes for PALF patients. [PPV 100% and NPV 58.2%, AUC 0.87 95% CI (0.74–0.95) *P* < 0.0001] (Fig. 2a).

### Prognostic scoring systems

Higher peak PELD/MELD scores were associated more strongly with adverse outcomes (*P* = 0.009) (Table 2) than admission PELD/MELD scores (*P* = 0.162). Although the association was not statistically significant, a one unit increase in PELD/MELD score at admission increased the risk of transplant or death by 10% [OR = 1.10, 95% CI (0.99–1.23), *P* = 0.073]. A cut off peak PELD/MELD score of greater than 29 using the ROC curve showed a sensitivity of 85% and specificity of 83% for poorer outcome [PPV 92% and NPV 70%, AUC 0.88 95% CI (0.75–0.96) *P* < 0.001] (Fig. 2b). Higher admission UKELD scores were also associated with death without transplantation (*P* = 0.010) as was KCHC (*P* = 0.002) (Table 2). When comparing all patients who recovered with their native liver and patients who died or received transplants, KCHC was statistically significant in predicting which patients would die without transplantation (*P* = 0.002) rather than predicting which patients would have poor outcomes (died or receive transplantation) or recover with their native liver (*P* = 0.084). A threshold of admission UKELD score greater than 63 showed a sensitivity and specificity of 80 and 83% respectively in the group of patients with poorer outcomes with a PPV and NPV of 92 and 65% respectively: AUC 0.89, 95% CI (0.76–0.96) *P* < 0.0001. (Fig. 2b) The proportion of Clichy scores was the same between the two groups of patients (*P* = 0.65). Higher LIU scores were observed in the group of patients who received liver transplantation (*P* = 0.019) (Table 2). We found that a liver injury unit score with a threshold of greater than 246 having a sensitivity of 84% and specificity of 83% [PPV 92% and NPV 70%, AUC 0.83 95% CI (0.69–0.92), *P* < 0.001]. (Fig. 2b) for predicting patients who died or required liver transplantation (poor outcomes).

**Table 2:** Scoring systems of pediatric patients referred with acute liver failure

Variable	Total ***N*** = 45 (100%)	Recovered (***n*** = 6)	Transplanted (***n*** = 20)	Died without transplant (***n*** = 19)	***P*** value
**Admission PELD score**					**0.162**
Mean (SD)(SD)	31.2 (11.2)	23.2 (11.6)	32.2 (10.8)	32.8 (10.9)	
**Peak PELD/MELD score**					**0.009**
Median (IQR))	43 (29–49)	25.5 (16–29)	47 (38.5–50)	45 (44–50)	
**LIU score**					**0.019**
Mean (SD)	429.5 (181.2)	251.4 (126.6)	483 (140.7)	429.3 (202.9)	
**Admission UKELD score**					**0.010**
Median (IQR)	66 (63–68)	62 (59–63)	66.5 (64–68)	66 (64–69)	
**KCHC fulfilled**					**0.002**
Yes	36 (80.0%)	3 (50.0%)	20 (100.0%)	13 (68.4%)	
**Peak UKELD score**					0.098
Mean (SD)(SD)	68 (64–69)	63.5 (59–65)	68 (67–69)	66 (64–69)	
**Time to Peak (days)**					**0.511**
Median (IQR))	2(1–2)	1.5 (0–2)	2 (1–4)	1 (1–2)	

*Abbreviations: PELD-* Pediatric end-stage liver disease, *MELD* Model for end-stage liver disease, *LIU* Liver injury Unit, *UKELD* United Kingdom end-stage liver disease score, *KCHC* Kings College Hospital Criteria, *IQR* Interquartile range

### Transplanted patients

Twenty patients (20/45; 44.4%) received liver transplantation. Patients with PALF in our cohort were appropriately referred to the transplant centre (*P* = 0.011), although there was no statistically significant difference in outcome between patients who were referred before and after February 2018 (*P* = 0.09) when the policy of direct referral to the transplant centre was introduced. Median times to presentation were higher in patients who were transplanted: 17 days (IQR 8–24) or died without transplantation: 14 days (IQR 7–22), than patients who recovered: {8.5 days (IQR 7–21)} (Supplementary figure). Thirteen patients (65%) received related living donor transplants (split) and the rest (7/20) were deceased donor liver transplants of which 2/7(28.6%) patients received the whole liver and the rest received split grafts (71%) (Supplementary table). Five (5/20; 25%) of the transplants were ABO incompatible transplants and were all performed after March 2018. Four transplanted patients (20%) demised within the first week post liver transplantation secondary to sepsis (1/5), haemorrhage (occult bleed) (1/5), fungal sepsis (1/5) and recurrent liver failure (1/5) respectively and one (5%) patient demised 16 months post transplantation from severe pneumocystis jiroveci pneumonia and acute respiratory distress syndrome while being treated for an acute rejection episode.

Nineteen (95%) recipients had medical complications after transplantation like CMV viraemia (6/20), sepsis (5/20), acute rejection (4/20), pleural effusion (2/20), acute respiratory distress syndrome (1/20) and pancytopenia (1/20) with 10/20 (50%) having surgical complications with biliary complications making up 6/10 (60%) of these complications. (Supplementary table). There was an 80% one-year patient and graft survival post liver transplantation for PALF patients in our cohort.

## Discussion

This observational single centre study evaluated the prognostic indicators associated with poorer outcomes in pediatric patients with ALF in Gauteng, S.A, which is currently the location of the only pediatric transplant unit in S.A, performing both living related and ABO incompatible liver transplantation in PALF patients [19]. To our knowledge this is the only study on prognostic factors and scoring systems in PALF from Southern Africa. In our cohort, viral etiology, most commonly HAV, was the predominant cause of PALF. This was consistent with a study done in Gauteng by Friedland et al., (Table 3) which found that 50% of children with ALF had an underlying diagnosis of HAV [34]. In S.A, HAV is not part of the routine immunization schedule. In HIC’s indeterminate etiology accounts for 40 to 50% of cases of PALF [8, 28].

**Table 3:** Studies summarizing pediatric acute liver failure studies from different countries

Authors	Year	Cohort size	Age	Location (income)	Etiology HAV/ Other viral (%)	Etiology Indeterminate /Other (%)	Etiology Autoimmune/metabolic (%)	Etiology Toxin/Reyes (%)	Prognostic Markers associated with poor outcome	Prognostic scores – poor outcome	Conclusion
Mlotha-Mitole et al. [20]	2021	24	1–13	S.A (Western Cape) Upper middle	16/20	29/17	8/0	12.5	INR > 4 Tot Bili > 210 IU/l		Viral causes predominate
Radaelli et al. [21]	2021	117		Central African Republic Low					INR > 4.55 ALT< 219 IU/l		PALF has significant prevalence
Bruckmann et al. [19]	2020	27	0–16	S.A Gauteng Upper middle	40.7/18.5	11/8	0/18	4			Transplant outcomes are good for PALF
Getsuwan et al. [22]	2020	27	0–16	Thailand Upper middle	0/30 (15% dengue infection)	25.9/11.1	0/14.8	22.2	Peak serum lactate > 6 mmol/L		Viral infections most common cause
Grama et al. [9]	2020	97	0–18	Romania High	0/19.6	11.3/8	10.3/14.4	36.8			Mortality higher in neonate/infants
Lee, Kim et al. [23]	2020	146	0–18	Korea High	3.4/6.8	47/14.3	3.4/15.7	8.9		Palf- Ds Peak Peld/Meld hdLIU	Palf-Ds superior to other scores
Mendizabal et al. [11]	2020	135	0–18	Argentina High	0/3.7	52/10.3	23/6	4	INR > 3.5, Bilirubin > 17 mg/dl		Risk staging model can be useful in predicting the need for transplant
Naveda-Romero et al. [24]	2020	44	0–14	Venezuela Unclassified	0/20.5	56.8/12.4	0/6.9	3.4	INR > 3.5	LIU > 240	Mortality 65.9% (high)
Nunez-Ramos et al. [25]	2018	20	0–15	Spain High	0/20	25/20	15/10	10		High PELD scores	PELD scores useful in predicting outcomes
Di Giorgio et al. [26]	2017	55	0-	Italy High	0/2	47/0	18/17	16	High bilirubin, INR, ammonia and low ALT		Survival of PALF > 90% with access to transplant
Tannuri et al. [12]	2016	115	0–18	Brazil Upper middle	16.5/5.2	54.8/1.7	9.6/9.6	2.6			Living donor outcomes for PALF are good
Ozcay et al. [27]	2016	91	0–18	Turkey Upper middle	25.3/7.7	33/5.5	1.1/12.1	15.4	High INR, Bilirubin, Lactate, ammonia	High PRISM and PELD scores in first 24 hours	Viral and indeterminate causes predominate Transplantation has high survival rates
Kathemann et al. [28]	2015	37	0–18	Germany high	8/8	43/8	8/13.5	11	High ammonia, low albumin and ALT on admission		Indeterminate causes predominate
Kaur et al. [29]	2013	58	0–18	India Low middle	58/18.4	9.2/0	4.6/10.2	0	Blood glucose < 45 mg/dlBilirubin > 10 mg/dl		Viral etiology is the commonest
Lu et al. [18]	2013	709	0–18	USA High	0/6.7	49.9/16.7	5.2/8.7	12.8	Liu superior to aLiu scores		Liu score is predictive of survival without liver transplant in PALF
Rajanayagam et al. [30]	2013	54	0–16	Australia High	0/15	69/9	6/26	13	INR > 4 Bilirubin > 220 IU/l	PELD/MELD at PALF diagnosis and peak	PELD > 27 at meeting PALF criteria and peak > 42 predictive of poor outcome
Sundaram et al. [31]	2013	522	0–18	USA High		43.1/				KCHC had a PPV 33% and NPV of 88% in predicting outcome	KCHC do not reliably predict that a child with non para PALF is likely to die if criteria are met (low sensitivity and PPV)
Sanchez et al. [32]	2011	50	0–18	Argentina High	42.5/0	35/0	17.5	5		Admission PELD/MELD	Admission PELD maybe helpful to predict liver transplant
Ciocca et al. [33]	2007	215	1–18	Argentina Upper middle	61/1.5	32/0.5	2.4/1	1.4	Bilirubin> 17 IU/ml INR > 4	KCHC specificity and sensitivity > 90%	KCHC useful in PALF where Hep A predominates as cause
Friedland et al. [34]	1991	17	0–13	S.A (Gauteng) Upper middle	35/29	5/0	0	29.4			

The World Health Organisation (WHO) guidelines suggest that improved sanitation, food safety and immunization are the most effective ways to combat HAV associated disease in our population [35]. Seroprevalence studies of HAV from S.A have in the past reported high endemicity with seroprevalence rates greater than > 90% in children up to 10 years of age [35, 36]. Endemicity of HAV in S.A varies by region and population groups with a recent shift in endemicity from high to intermediate endemicity for HAV infection in areas with increased urbanisation and improved sanitation [37] with the average age of HAV shifting from children to older age groups [35]. One of the possible reasons for this is the dichotomous healthcare and patient population in S.A [38] including a self-funded private sector patient population and public sector patients funded by government. The private sector population group mimics high-income countries with decreased seroprevalence of HAV and although HAV immunisation is currently not part of the expanded programme of immunization in S.A, routine vaccination is recommended in this group of patients [35, 36]. Revisiting of HAV immunisation policies in S.A are necessary and would contribute to modifying the etiology and occurrence of PALF.

Biochemical parameters, both in isolation and by incorporation into prognostic scoring systems, are important prognosticators of outcome in PALF patients. Kathemann [28] et al., Di Giorgio [26] et al. and few other studies [22, 39] found significantly higher INR, peak bilirubin and peak ammonia levels in PALF patients with poorer outcomes, as with our cohort of patients. Higher peak lactate [22, 40] has also been described in some studies as predictor of poor outcomes in PALF patients and has especially been studied as a component of KCHC in paracetamol-associated PALF patients.

Kings College Hospital Criteria (KCHC), admission PELD/MELD and Clichy scores, although utilized often in our setting to determine referral to the transplant center, were less helpful than the liver injury unit, admission UKELD and peak PELD/MELD scores to determine which patients would have poorer outcomes. We found KCHC more useful in predicting which patients required liver transplantation than which patients would die if criteria were met. This was consistent with a study published by Sundaram et al. [31]. Although the PELD/MELD and UKELD scoring systems are used as predictors of mortality in children with chronic liver disease, Sanchez [32] and Nunez-Ramos et al. [25] found admission PELD/MELD levels to be significantly higher among children with poor outcomes from PALF (Table 3). Our findings were that peak PELD/MELD was superior to admission PELD/MELD scores for predicting poor outcomes as also reported by Rajanayagam et al. (Table 3) who found that serial PELD/MELD scores were more useful in predicting outcomes [30].

The Liver injury units is a scoring system which has shown to be predictive of survival without liver transplantation in a single center retrospective analysis by Lu et al. (Table 3) who demonstrated this score to have a high specificity and sensitivity for predicting death/liver transplantation [18]. This correlates with our findings and that PALF is a dynamic process requiring regular clinical and biochemical assessments of patients to ensure optimal management and prevent unnecessary transplantation in a setting where the patient would recover with supportive management. A disadvantage of this scoring system is that it is accurate at predicting (poor outcomes) death or liver transplant, not death alone from PALF [15, 16]. This scoring system is not currently in clinical use but a recent study done by Naveda-Romero et al. [24] in pediatric patients in Venezuela found a LIU score of greater than 240 to be associated with poorer outcomes which correlated with findings from our cohort of patients (Table 3).

Liver transplantation has interrupted the clinical trajectory of PALF [17, 41]. In South Africa’s heterogenous population, access to transplantation is limited and dependent on many factors like socio-economic status, geographical location, access to healthcare, transportation availability to health care facilities and many other factors [19]. In our cohort there were many patients who, although referred for transplantation, were too ill to be transplanted. This reflects an increasing need for community and health education programs to encourage earlier referral. Our center is fortunately near a transplant center and PALF patients referred to us have access to transplantation. Direct referral to our transplant center did not significantly impact outcomes of PALF but this finding was expected as pre-transplant management of PALF in our center and referring units, follow the same principles as the transplant center, allowing timeous waitlisting of PALF patients. With the paucity of deceased donation in S.A, related living donation and ABO incompatible transplantation [42] at our transplant center has resulted in an improvement in the access to liver transplantation in PALF patients.

### Limitations

Limitations of this study was that it was a retrospective, single center study with a small number of patients in the cohort and therefore lacked generalizability. We were reliant on note taking and unavailability of information would have affected certain variables and analysis. Retrospectively analysing biomarkers and scoring systems which may have been used to decide on liver transplantation introduced inherent bias, and it is unknown whether all patients who received a liver transplant would have demised without it. The actual number of patients with PALF in our setting is largely unknown and not all eligible children with PALF were referred for transplantation to our center or had access to pediatric intensive care units. These deficits in the system need to be addressed at a national government level so that adequate solutions can be sought.

## Conclusion

PALF, although uncommon, remains a devastating illness in previously well children [1, 2, 4, 23]. Prognostic markers and scoring systems currently utilized to assess outcome are largely extrapolated from adult studies [15, 16]. Findings in our study showed an increased number of patients who died prior to liver transplantation compared with other high and low-income countries [21, 22, 28, 29]. Although our study demonstrates the utility of dynamic scoring systems in PALF patients, it underscores the need for early referral and clinical monitoring in a tertiary center once the criteria for PALF have been met. S.A would also benefit from multi center registries [6, 7, 10] to assist in formulating and standardizing scoring systems that could be utilized to best manage this group of patients.

## Supplementary Information

**Additional file 1.**

## Data Availability

The datasets used and/or analysed during the current study are available from the corresponding author on reasonable request.

## Abbreviations

ALF: Acute liver failure
HAV: Hepatitis A virus
HIC: High income country
INR: International normalised ratio
KCHC: Kings College Hospital Criteria
LMIC: Low-middle income country
LIU: Liver injury unit
MELD: Model for end-stage liver disease
NPV: Negative predictive value
PALF: Paediatric acute liver failure
PELD: Paediatric end-stage liver disease
PPV: Positive predictive value
ROC: Receiver operating characteristic
UKELD: United Kingdom end-stage liver disease
