# Supplementary material for: Prognostic factors and scoring systems associated with outcome in pediatric acute liver failure
Source: BMC Pediatr. 2022 Aug 31;22:516. doi: 10.1186/s12887-022-03574-x (PMC9429365; doi:10.1186/s12887-022-03574-x)
Supplement: Supplementary file 1 — Additional file 1. [file 12887_2022_3574_MOESM1_ESM.docx]

**SUPPLEMENTARY TABLES**

Table 1. Liver transplantation in pediatric acute liver failure patients referred to Charlotte Maxeke Johannesburg Academic Hospital (tertiary academic hospital in South Africa)

| Variable | N (%) | N (%) |  |
| --- | --- | --- | --- |
| ABO blood type incompatible |  |  |  |
| Yes | 5 (11.1) |  |  |
| Received transplant |  |  |  |
| No | 25 (55.6) |  |  |
| Spontaneous recovery native liver | 6 (24) |  |  |
| Died – not transferred to referral centre or transplant centre as too unstable | 5 (20) |  |  |
| Died - Listed but not transplanted as demised prior to transplant | 5 (20) |  |  |
| Died – transferred to transplant centre but died prior to listing | 9 (36) |  |  |
| Yes (Type): | 20 (44.4) | Mortality 5/20 (25) |  |
| Deceased donor | 7 (35.0) | 3/7 (43) |  |
| Related living donor | 12 (60.0) | 1/12 (8) |  |
| Related living donor (HIV discordant) | 1 (5.0) | 1/1 (100) |  |
| Histology of explant* |  |  |  |
| Massive necrosis | 14 (70.0) |  |  |
| Sub-massive necrosis | 4 (20.0) |  |  |
| Bridging necrosis | 1 (5.0) |  |  |
| Lymphoma | 1 (5.0) |  |  |
| Medical complications* |  |  |  |
| Yes | 19 (95.0) |  |  |
| Type of surgical complication* |  |  |  |
| No surgical complications | 10 (50.0) |  |  |
| Bile leak | 1 (5.0) |  |  |
| Biliary stricture | 3 (15.0) |  |  |
| Biliary stricture + liver abscess | 1 (5.0) |  |  |
| Biliary stricture + necrotic bowel | 1 (5.0) |  |  |
| Hepatic artery thrombosis | 1 (5.0) |  |  |
| Open abdomen | 2 (10.0) |  |  |
| Small for size syndrome | 1 (5.0) |  |  |

**SUPPLEMENTARY FIGURES**

**FIGURE 1**

**Boxplot of median times to presentation(days) in all PALF patients referred to Charlotte Maxeke Johannesburg Academic Hospital**


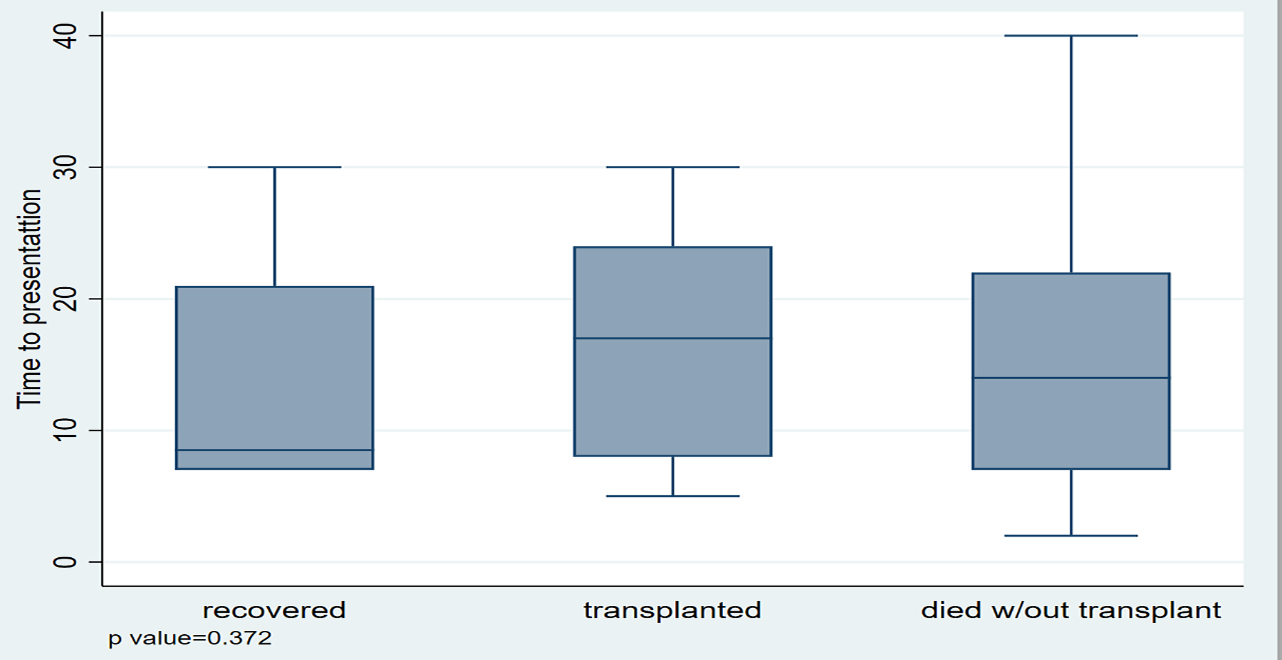


Figure 1. Median time to presentation of PALF patients referred to Charlotte Maxeke

Johannesburg Academic Hospital
